# Community-level correlates of physical violence against unmarried female adolescents in Bangladesh

**DOI:** 10.1186/1471-2458-14-1027

**Published:** 2014-10-02

**Authors:** Kristin VanderEnde, Sajeda Amin, Ruchira Tabassum Naved

**Affiliations:** International Centre for Diarrhoeal Disease Research, Bangladesh, (icddr,b), Dhaka, Bangladesh; Program on Poverty, Gender, and Youth, Population Council, New York, USA; Center for Equity and Health Systems, International Centre for Diarrhoeal Disease Research, Bangladesh (icddr,b), Dhaka, Bangladesh

**Keywords:** Bangladesh, Violence against women and girls, Adolescents, Community characteristics

## Abstract

**Background:**

While the majority of research in Bangladesh has focused on intimate partner violence (IPV) against women, less is known about the correlates of physical violence against unmarried female adolescents, particularly community-level characteristics that may increase their risk of experiencing violence.

**Methods:**

We used multilevel logistic regression analysis to assess the variability in physical violence against unmarried female adolescents at the community level and to explore the role of community-level characteristics in explaining this variability. The data for this analysis were taken from a 2005 nationally representative survey of 20,000 adolescents aged 10 to 24 living in Bangladesh. Data from 4,370 unmarried female adolescents were included in the final model.

**Results:**

Communities in Bangladesh have, on average, high levels of physical violence against unmarried female adolescents, and these levels vary widely across communities. Community-level acceptance of physical punishment against adolescents was related to unmarried female adolescents’ risk of experiencing physical violence.

**Conclusions:**

It is important to find and target communities in which unmarried adolescent girls are at higher risk of experiencing physical violence. Programs and policies must focus specifically on changing attitudes regarding treatment of women and girls. As these attitudes accepting of physical violence are found in adolescents aged 10 to 19, school and community-based programs should particularly target this age group.

## Background

In Bangladesh, violence against women and girls (VAWG) is common [1, 2] and has serious health consequences [1–7]. While the majority of research on violence against women and girls in Bangladesh has focused on intimate partner violence (IPV) experienced by married women [8–10], there is a growing recognition of the problem of violence experienced by unmarried adolescent girls [11, 12]. In Bangladesh, a patriarchal society, VAWG is often considered to be acceptable behaviour [13, 14]. Girls and young women are the most common victims of acid attacks [15], and are a high-risk group for experiencing violent death [16]. In Bangladesh, where adolescents between the ages of 10–19 compose one-fourth of the total population [17], and physical punishment of adolescents is common and acceptable [12, 18], factors such as low educational levels and physical violence increase the vulnerability of adolescent girls [18]. Despite the magnitude of the problem of violence against adolescent girls in Bangladesh, there is a scarcity of resources available to address this important public health issue. Thus, it is important to identify geographic variation in this violence and the community-level characteristics that place adolescent girls at increased risk. Once high-risk communities and community-characteristics are identified, interventions can be targeted specifically to focus on areas with the greatest need.

### Community context

An ecological framework [19] characterizes VAWG as consequence of direct and interacting influences operating at the individual, relationship, family, community, and societal levels. Historically, the majority of research on VAWG has focused on individual, relationship, and household characteristics, but in the past decade, an increasing amount of research has highlighted the influence of community characteristics on health outcomes, including IPV against women [20] and child maltreatment [21]. The majority of researchers in these areas have drawn from social disorganization theory and focused on the relationship between aggregate measures of socioeconomic status, such as levels of poverty or levels of education, and risk of violence or abuse [20]. Other research has examined the relationship between community norms regarding the acceptability of violence, the social connectedness of a community, or particularly for violence against women, community norms regarding gender roles [20, 21]. In India, for example, high levels of community norms accepting wife-beating have been associated with higher levels of male perpetration [22] and women’s reported experience of IPV [23]. Additionally, in Bangladesh, many of the associations between individual, relationship, and household factors and physical violence against married women have been shown to vary across communities [9]. For example, Koenig and colleagues [9] found that for women living in a culturally conservative area of Bangladesh, individual membership in short-term credit groups and high individual women’s autonomy were associated with an increased risk of violence, but community characteristics were not significant. In a less conservative area of Bangladesh, however, both community-level measures of women’s group membership and high women’s autonomy were associated with a lower risk of violence, while individual-level credit group membership and autonomy were not significantly related to IPV [9]. These findings suggest that the relationship between women’s autonomy, their membership in microcredit groups, and their experiences of violence may be context dependent. In culturally conservative areas, these behaviors may be seen as challenging traditional gender norms and may place women at increased risk of experiencing violence [9]. In summary, these findings highlight the importance of identifying community-level characteristics that place women and adolescent girls at greatest risk of experiencing violence. Once identified, these research findings can be used to design and implement interventions specifically targeting these community-level characteristics.

## Methods

In this analysis, we assessed community-level variability in physical violence against unmarried adolescent girls and explored the role of community-level characteristics in explaining this variability. We drew from feminist perspectives, which view VAWG as stemming from inequality between men and women both within the home and outside of it [24]. We posited that community characteristics that have been explored in relation to IPV against married women are relevant to explain community-level variation in physical violence against unmarried female adolescents in Bangladesh. In addition, we assessed the variability in the prevalence of physical violence against unmarried female adolescents at the community level, and explored the role of community-level characteristics, such as poverty, female literacy, attitudes towards violence, the percentage of households belonging to a non-governmental organization (NGO), and degree of urbanization in explaining this variability. We also examined whether the relationships between physical violence against adolescent girls and household- and community-level NGO membership varied across communities with different levels of acceptance of physical violence against adolescents.

The data for this secondary analysis were taken from a 2005 nationally representative survey of adolescents aged 10 to 24, both unmarried and married, living in Bangladesh. A sampling frame provided by the Bangladesh Bureau of Statistics was used to draw the sample. The study, which surveyed approximately 20,000 homes, was conducted in the same Primary Sampling Units (PSU) as the 2003-4 Bangladesh Demographic and Health Survey (DHS) [13]. The study employed a two-stage probability proportional to size (PPS) sampling technique, with each administrative division stratified into rural and urban areas. A total of 361 PSUs were randomly selected; 277 in rural areas and 84 in urban areas.

Each PSU contained approximately 200 households. Sixty households were randomly selected from each cluster following a systematic random sampling method. In this analysis, we measured community-level variables at the PSU-level, a method consistent with the global literature on communities and IPV against women [20, 23, 25–27]. A total of 20,000 households were targeted for participation in the survey. Household information was collected from each selected household within a PSU. The Kish method was used to randomly select one adolescent per household for participation in the survey. A total of 14,942 adolescents aged 10–24 were selected for the initial study. Of these, 4,370 unmarried female adolescents between the ages of 10–19 were included in the present analysis.

The original study applied a strict set of ethical guidelines and maintained the confidentiality and anonymity of the research participants [28]. Ethical approval for the original study was obtained from the scientific team of BRAC Research and Evaluation Division. Administration of the household questionnaire preceded interviews of the adolescents. Informed consent was obtained from the parents/guardians before each household interview. Then assent/consent was obtained from each adolescent participating in the study. The current secondary analysis used only de-identified data. icddr,b’s Ethical Review Committee approved the study.

### Measures

#### Outcome variable

*Any physical violence* against unmarried female adolescents was represented by unmarried adolescent girls aged 10–19 reported response (yes = 1, no = 0) to the question “did anyone hit or beat you in the past year?”. Seven respondents providing no response were dropped from the final analysis.

### Community-level variables

#### Wealth ranking

Following the methodology of Filmer and Pritchett [29], we constructed a household wealth index using information on ownership of household assets, land, and self-assessed measures of sufficiency of food and clothing from the household questionnaire. Considering the differences in types of assets and living standards between urban and rural areas, we used separate rankings for urban and rural measures. To reflect a focus on differences between economic status in poorer, compared to non-poor communities, we created aggregate-level measures by ranking the mean household wealth index for each community, and then created a dichotomous measure by coding the lowest 40% of communities poor (coded as 0, the reference category), and the highest 60% as non-poor (coded as 1). This approach is consistent with research examining community-level economic status and IPV against women [20].

#### Female literacy

To construct a measure of levels of female adult literacy per community, we used information from the household questionnaire. The measure of female adult literacy reflected the percentage of adult women (>19 years old) in each community or district who stated they could “read and write [a] letter”.

#### Acceptance of physical punishments of adolescents

We operationalized a measure of community-level acceptance of physical punishment against adolescents as the percentage of unmarried adolescents, both male and female, ages 10–19 agreeing that children ages 12 and older “should be beaten up when they do an offence, e.g. stealing, telling a lie, running away from school”.

#### Households belonging to an NGO

Using information from the household questionnaire, we measured households belonging to an NGO as the percentage of households currently belonging to an NGO for each community. Households reporting membership in any NGO (including, but not limited to, microcredit organizations) were coded 1, and households reporting they were not a member of any NGO were coded 0, the reference category.

#### Degree of urbanization

At the community-level, we included a measure for degree of urbanization. Urban communities were coded 1, and rural communities were coded 0, the reference category.

### Control variables

#### Individual controls

At the individual level, we included controls for age (in years) and religion (Muslim = 1, other = 0). *Family/household controls* included a measure for household membership in an NGO (yes = 1, no = 0). We constructed a household wealth index as described previously for community wealth. We coded the lowest 40% of households as 0 (reference category), and the highest 60% of households as 1.

### Analysis

We ran univariate descriptive statistics on all variables, assessing for missing values and normal distributions. We estimated bivariate associations between all covariates to assess potential co-linearity. Next, we examined the relationship between characteristics of communities and an individual female adolescent’s risk of experiencing any physical violence, controlling for factors at the individual, family, and household levels. Following the survey design, in which communities were clustered within districts, we accounted for variation between districts in our analysis, although we did not include any predictors at the district level. We employed three-level logistic models predicting an unmarried female adolescent’s likelihood of experiencing physical violence. These outcomes were measured dichotomously, with y = 1 if she reported an experience of violence, and y = 0 otherwise. We estimated an unconditional means model to estimate variation in physical violence between communities and districts (tau). Next, we added variables at the community and individual levels, first estimating models with a single community-level covariate, controlling for individual-level covariates, (not shown), then a model with all community-level covariates.

We grand-mean centered individual- and community-level variables, allowing for the interpretation of community-level coefficients controlling for individual-level variables. We first constrained the slope of each individual- and community-level covariate to be the same fixed value for each community- and district-level unit. In a subsequent step, we added separate interactions between community-level acceptance of physical punishment of adolescents and household- and community-level NGO membership. We compared models including weights to adjust for the probability of selection at the individual-level to un-weighted models, but no difference was found, therefore, the results presented represent un-weighted analyses. We employed adaptive Gaussian estimation techniques in HLM 7, which are appropriate for parameter estimation in models with binary outcomes [30].

## Results

### Characteristics of sample

Socio-demographic characteristics of unmarried female adolescents including age, household wealth, NGO membership, and religion, along with reported prevalence of physical violence, are presented in Table 1. On average, unmarried female adolescents were 13.3 years old, and from predominantly Muslim households (86.1%). Over one-third (33.0%) of unmarried adolescents reported living in a household that belonged to an NGO. A significant proportion of unmarried female adolescents (37.9%) reported experiencing physical violence in the past year (Table 1).

**Table 1:** **Characteristics of sample**

	Unmarried female adolescents ^a^
	n = 4377
**Any past year physical violence**	
Yes	1661 (37.9%)
*Missing* ^*b*^	*7 (0.2%)*
**Age in years, mean (SD)**	13.3 (2.5)
**Household wealth**	
Non- poor (highest 60%)	2,679 (61.2%)
**Religion**	
Muslim	3770 (86.1%)
**NGO membership**	
Yes	1445 (33.0%)

^a^Unmarried adolescents aged 10–19 years.

^b^Respondents with missing data were excluded from multi-level logistic regression models.

### Community characteristics

Community characteristics are presented in Table 2. The percentage of unmarried female adolescents in each community reporting any physical violence in the past year ranged from 0-100%, with a mean of 38.5%. The mean community-level adult female literacy was 36.6%, but this varied widely between communities (Table 2). Similarly, there were wide ranges in the percentage of adolescents reporting attitudes accepting of physical punishment at the community level (9–95%). On average, 32.1% of households in a community belonged to an NGO, but this varied widely between communities (0 - 78%). The majority of communities (76.7%) were from rural areas (Table 2).

**Table 2:** **Characteristics of communities**

	Communities (n = 361)	
	***Mean (SD)***	***Range***
% unmarried female adolescents reporting any past year physical violence	38.5 (22.4)	0 - 100
% literate (adult females)	36.6 (17.7)	5 - 92
% accepting of physical punishment of adolescents	50.6 (17.5)	9 – 95
% households belonging to an NGO	32.1 (16.0)	0 - 78
Wealth ranking (n,%)	*n*	*%*
Non-poor (highest 60%)	217	60.1%
Area (n,%)		
Rural	227	76.7%

### Multi-level analyses

Results of the multi-level logistic models predicting an unmarried female adolescent’s likelihood of experiencing physical violence are presented in Table 3. At the individual level, we found a negative association between age and physical violence, but no relationship between religion, household wealth, or household membership in an NGO and the outcome. At the community level, we found a strong positive association between community-level acceptance of physical punishment of adolescents and any physical violence against unmarried female adolescents (aOR 5.42, 95% CI 2.52-11.64). We found no relationship between the percentage of households belonging to an NGO, community wealth, female literacy, and degree of urbanization and past year physical violence in models that control for age, religion, poverty and NGO membership at the household level (Table 3).

**Table 3:** **Multi-level logistic models for any physical violence against unmarried female adolescents in Bangladesh**

	aOR (95% CI) Unmarried female adolescents
	n = 4370
Intercept	0.50 (0.41-0.61)*
**Community-level variables**	
Community wealth (poor ref)	
Non-poor	1.28 (0.96-1.72)
% literate (female)	0.68 (0.26-1.79)
% accepting of physical punishment of adolescents	5.42 (2.52-11.64)*
% households belonging to NGO	1.99 (0.88-4.54)
Area (rural ref)	
Urban	0.98 (0.69-1.39)
**Individual-, family-, and household variables**	
Age	0.65 (0.63-0.68)*
Religion (non-Muslim ref)	
Muslim	1.20 (0.91-1.58)
Household wealth (poor ref)	
Non-poor	1.00 (0.84-1.19)
NGO membership (non-membership ref)	
Household member of NGO	1.05 (0.88-1.25)
**Random effects** tau (SE)^†^	
Community level	0.53 (0.10)
District level	0.43 (0.12)

*p < 0.01, ^†^Significance of random effects not estimated.

## Discussion

Results of this study indicate that Bangladesh is characterized by high levels of physical violence against unmarried female adolescents, but this varies widely across communities. Specifically, our analysis found that community-level acceptance of physical punishment against adolescents was related to adolescent girls’ risk of experiencing physical violence after controlling for individual, family, and household factors. These finding are consistent with the broader literature on violence against women, which suggests that community-level acceptance of forms of violence against women, such as wife-beating, is a strong predictor of women’s experience of IPV [23], or male perpetration of IPV [22]. These findings highlight the importance of community context, particularly community norms accepting violence, in regards to physical violence against women and girls, and also have implications for public health programmes and policy. Programmes and policies aimed at reducing levels of physical violence against unmarried adolescent girls must address community norms accepting of violence in the design and implementation of programmes targeting these populations. Interventions aimed at challenging the acceptability of violence against women and girls, such as school-based programmes or media campaigns, may be an appropriate first step.

The non-significant finding for community-level wealth is consistent with findings from multi-level studies of IPV against women in non-Western settings, which have not demonstrated a relationship between community-level standard of living and IPV against women [20, 23, 25]. This finding, along with the non-significant finding for degree of urbanization, suggests that future research should focus on more relevant community characteristics, particularly the social aspects of communities such as norms and attitudes accepting of violence, in relation to physical violence against adolescent girls. We did not find a relationship between the percentage of literate females in a community and physical violence against unmarried female adolescents. These results contrast with literature indicating a protective effect of women’s education on spousal violence [26, 31], suggesting more research is needed to clarify these relationships. Lastly, we did not find a significant direct relationship between household- or community-level NGO membership and physical violence against unmarried female adolescents, nor did we find any significant interactions between household- or community-level NGO membership and community acceptance of physical punishment of adolescents. Future research should continue to explore these relationships, as a higher level of community involvement in NGOs, which often focus on poverty alleviation, was hypothesized to mitigate levels of violence against unmarried female adolescents, particularly in communities where physical violence is not condoned. Similarly, in cross-sectional, retrospective research in Bangladesh, women’s membership in microcredit programmes has been associated with both increases and decreases in risk of IPV against women [9, 32, 33]. There is a pressing need for longitudinal research to further explicate these relationships, particularly changes in trajectories of risk of experiencing physical violence over time.

The findings of the study have several limitations. First, the cross-sectional design of our study does not allow for causal inference. Secondly, we analysed data from a general survey of adolescents, and our measure of physical violence was limited to the responses to a single question about past year experiences of physical violence. Our measure did not assess frequency or severity of violence, did not capture other forms of violence such as emotional or sexual violence, and did not account for the type of perpetrator or multiple perpetrators. Thirdly, no data were available on whether the respondent witnessed intimate partner violence in the home, a noted risk factor for both childhood physical abuse and IPV against women [10, 34]. These limitations highlight the need for population-based surveys focused specifically on violence against unmarried adolescent girls in Bangladesh. Recent national surveys of childhood physical and sexual violence, conducted primarily in Sub-Saharan Africa [35–37], capture a broad range of information on the prevalence and associated health consequences of violence in childhood and adolescence, and may offer a model for future research in Bangladesh.

## Conclusions

This study, to our knowledge, is the first to describe community-level correlates of physical violence against unmarried adolescent girls in Bangladesh and suggests important directions for public health programmes and policy. Due to overall high levels of physical violence against unmarried adolescent girls, and variation in these levels across communities, it is important to find and target communities in which adolescent girls are at higher risk. At the community level, high levels of acceptance of violence may girls at risk. Programmes and policies must focus specifically on changing attitudes regarding treatment of women and girls, particularly in districts with the highest levels of violence. As these attitudes accepting of physical violence are found in adolescents aged 10 to 19, school and community-based programmes should particularly target this age group.

## Abbreviations

HLM: Hierarchical linear modelling
IPV: Intimate partner violence
NGO: Non-governmental organization
PSU: Primary sampling units
PPS: Probability proportional to size
SD: Standard deviation
SMA: Statistical metropolitan areas
VAWG: Violence against women and girls.
